# Evaluating large language models for health-related text classification tasks with public social media data

**DOI:** 10.1093/jamia/ocae210

**Published:** 2024-08-09

**Authors:** Yuting Guo, Anthony Ovadje, Mohammed Ali Al-Garadi, Abeed Sarker

**Affiliations:** Department of Biomedical Informatics, Emory University, Atlanta, GA 30322, United States; Department of Biomedical Engineering, Georgia Institute of Technology, Atlanta, GA 30332, United States; Department of Biomedical Informatics, Vanderbilt University, Nashville, TN 37235, United States; Department of Biomedical Informatics, Emory University, Atlanta, GA 30322, United States; Department of Biomedical Engineering, Georgia Institute of Technology, Atlanta, GA 30332, United States

**Keywords:** text classification, large language models, natural language processing

## Abstract

**Objectives:**

Large language models (LLMs) have demonstrated remarkable success in natural language processing (NLP) tasks. This study aimed to evaluate their performances on social media-based health-related text classification tasks.

**Materials and Methods:**

We benchmarked 1 Support Vector Machine (SVM), 3 supervised pretrained language models (PLMs), and 2 LLMs-based classifiers across 6 text classification tasks. We developed 3 approaches for leveraging LLMs: employing LLMs as zero-shot classifiers, using LLMs as data annotators, and utilizing LLMs with few-shot examples for data augmentation.

**Results:**

Across all tasks, the mean (SD) *F*_1_ score differences for RoBERTa, BERTweet, and SocBERT trained on human-annotated data were 0.24 (±0.10), 0.25 (±0.11), and 0.23 (±0.11), respectively, compared to those trained on the data annotated using GPT3.5, and were 0.16 (±0.07), 0.16 (±0.08), and 0.14 (±0.08) using GPT4, respectively. The GPT3.5 and GPT4 zero-shot classifiers outperformed SVMs in a single task and in 5 out of 6 tasks, respectively. When leveraging LLMs for data augmentation, the RoBERTa models trained on GPT4-augmented data demonstrated superior or comparable performance compared to those trained on human-annotated data alone.

**Discussion:**

The results revealed that using LLM-annotated data only for training supervised classification models was ineffective. However, employing the LLM as a zero-shot classifier exhibited the potential to outperform traditional SVM models and achieved a higher recall than the advanced transformer-based model RoBERTa. Additionally, our results indicated that utilizing GPT3.5 for data augmentation could potentially harm model performance. In contrast, data augmentation with GPT4 demonstrated improved model performances, showcasing the potential of LLMs in reducing the need for extensive training data.

**Conclusions:**

By leveraging the data augmentation strategy, we can harness the power of LLMs to develop smaller, more effective domain-specific NLP models. Using LLM-annotated data without human guidance for training lightweight supervised classification models is an ineffective strategy. However, LLM, as a zero-shot classifier, shows promise in excluding false negatives and potentially reducing the human effort required for data annotation.

## Introduction

Social media platforms serve as a valuable medium for patients to share and discuss their health-related information, encompassing a broad spectrum of topics.^1–3^ To derive knowledge about these topics, researchers have employed natural language processing (NLP) technologies,^4^^,^^5^ often employing text classification methods. Supervised classification of social media data is particularly challenging, relative to texts from other sources, due to the inherent noise. The linguistic characteristics of the text can vary significantly depending on the originating social media platform. For instance, Twitter (rebranded as X) posts commonly feature hashtags and emojis, whereas this is not typical for posts on Reddit. Another challenge in text classification for health-related tasks involving social media data lies in the effectiveness of data collection due to the presence of non-standard expressions, misspellings, and noise.^6^^,^^7^ The traditional data collection pipeline for social media comprises 2 key steps: (1) filtering out unrelated posts using a keyword list, and (2) manually annotating the selected posts. Given that most social media content is typically unrelated to the research topic of interest, the resultant dataset often exhibits a markedly reduced size, or the class distribution tends to be highly unbalanced.

Pretrained language models (PLMs), such as BERT^8^ and RoBERTa^9^, have demonstrated remarkable success in a wide range of NLP tasks. Encouraged by the success of transformer-based PLMs, many studies have focused on adapting them to text classification tasks involving social media data. Nguyen et al^10^ proposed BERTweet by pretraining a transformer-based model from scratch on a large set of English posts from Twitter. Guo et al^11^ developed SocBERT, which was pretrained on English posts from Twitter and Reddit. Qudar and Mago^12^ developed TweetBERT by continuing pretraining the language model of BERT. Additionally, research efforts have been made to organize shared tasks or competitions for text classification of health-related topics in social media. For example, the Social Media Mining for Health (SMM4H) shared tasks have covered a wide range of health-related topics over the years, including pharmacovigilance, toxicovigilance, and involved data from different social media platforms, such as Twitter and Reddit.^13–17^ Additionally, the Sixth Workshop on Noisy User-generated Text (W-NUT 2020) proposed a text classification task aiming to identify informative COVID-19 English posts from Twitter.^18^

Although the PLMs have achieved success, the model performance is highly dependent on the quality and quantity of annotated data of the downstream tasks. Powell et al^19^ demonstrated that conventional machine learning models surpassed a PLM in classifying fall types among individuals with Parkinson’s disease. The authors posited that the limited dataset size and class imbalance were suboptimal for PLM performance in this context. Similarly, Guo et al^20^ reported comparable findings when employing a PLM to identify Fontan patients using clinical notes, which contained substantial noise. These studies highlight potential limitations of PLMs in certain healthcare applications, particularly when dealing with restricted or imbalanced datasets. Researchers have made attempts to reduce the need for annotated data by pretraining larger language models (LLMs) with a generative model architecture such as GPT3.^21^ In 2023, a chatbot named ChatGPT, powered by an LLM named GPT3.5 with 175B parameters and pretrained on a large corpus of text data from the Internet, achieved great success on various question-answering NLP tasks. Kung et al^22^ showed that ChatGPT performed at or near the passing threshold for the United States Medical Licensing Exam without requiring supervision with human-annotated data. Chen et al^23^ examined the performance of ChatGPT on various neurological exam grading scales, where ChatGPT demonstrated ability in evaluating neuroexams using established assessment scales. Similarly, Dehghani et al^24^ evaluated the performance of ChatGPT on a radiology board-style examination, and ChatGPT correctly answered 69% of questions. Despite their primary purpose as generative models for text generation, LLMs have been effectively utilized in text classification endeavors. Some studies have directly tasked LLMs with predicting classifications in a zero-shot setting, a machine-learning approach that functions without prior training data.^25–30^ Additionally, research has delved into investigating techniques for integrating LLMs into text classification tasks, such as leveraging them for data augmentation^31–33^ and using LLMs to obtain external knowledge.^34^^,^^35^

Inspired by the success of LLMs, we explore strategies for leveraging them for social media-based health-related text classification. Potentially, via effective zero-shot classification, LLMs may significantly diminish the time and cost associated with data annotation. In this work, we sought to explore this potential by leveraging LLMs in 3 different settings: employing LLMs as zero-shot classifiers, using LLMs as annotators to annotate training data for supervised classifiers, and utilizing LLMs for data augmentation. Our contributions are outlined below:

- We developed and compared 3 approaches for integrating LLMs into text classification.
- We conducted a comprehensive benchmarking exercise, evaluating 1 supervised classic machine learning model based on Support Vector Machines (SVMs),^36^ 3 supervised PLMs (RoBERTa,^9^ BERTweet,^10^ and SocBERT^11^), and 2 zero-shot classifiers based on GPT3.5^37^ and GPT4,^38^ across 6 text classification tasks.
- Our findings demonstrate that the most optimal strategy is to leverage in-context trained LLMs for augmenting human-annotated data. Nevertheless, future investigations are needed to explore the determination of appropriate training data size and the optimal volume of augmented data.

## Methods

### Data collection

In this work, we conducted 6 text classification tasks covering diverse healthcare topics with data from Twitter. Among these tasks, 4 tasks—classification of self-report breast cancer, classification of change in medications regimen, classification of self-report of adverse pregnancy outcomes, and classification of self-report potential cases of COVID-19—are from Social Media Mining for Health Applications (SMM4H) shared tasks,^4^ and 2 tasks—classification of self-report depression and self-report Chronic Obstructive Pulmonary Disease (COPD)—are private data sets collected and annotated by our team. To collect self-reported tweets related to depression and COPD, we identified the tweets containing specific phrases that indicate personal experiences such as “I feel depressed” and “I have COPD.” The data were manually annotated by 2 reviewers, and the annotation process achieved inter-annotator agreement percentages of 0.84 for self-report depression and 0.87 for self-report COPD. All of the tasks are binary classification, and the evaluation metrics are precision, recall, and *F*_1_ score for the positive class. The data statistics of the tasks are shown in Table 1. The sample positive and negative tweets for each classification task are listed in Table S1.

**Table 1.** The number of positive and negative classes and the data size for 6 classification tasks.

Task	Positive	Negative	Total
Self-report depression	625	477	1102
Self-report COPD	401	373	774
Self-report breast cancer	1283	3736	5019
Change in medication regimen	656	6814	7470
Self-report adverse pregnancy outcomes	2922	3565	6487
Self-report potential cases of COVID-19	1148	6033	7181

### Classification models

**Figure 1.**
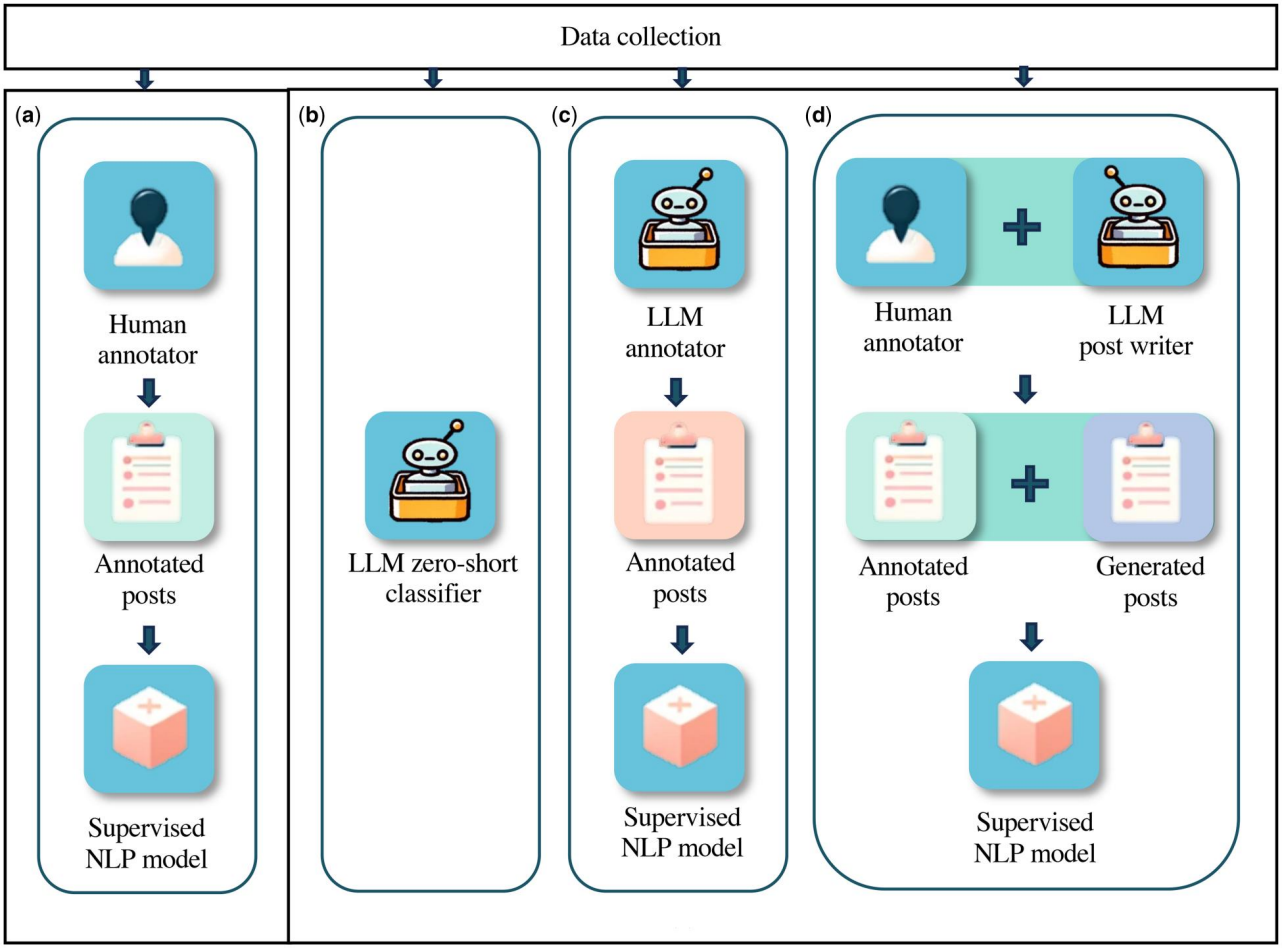
The framework for comparing 4 supervised text classification strategies for benchmarking: (A) standard supervised classification approach with manually annotated data; (B) employing the LLM as a zero-shot classifier; (C) using the LLM as an annotator for data that can be used by supervised classification approaches; and (D) utilizing LLMs for data augmentation for traditional supervised classification models.

The overall classifier benchmarking framework of this study is shown in Figure 1. We developed the supervised NLP models, including 1 supervised classic machine learning model based on SVMs, and 3 supervised PLMs (RoBERTa, BERTweet, and SocBERT). We refer to these as *traditional* supervised classification approaches. We also developed 3 approaches for integrating LLMs into text classification. These methods include employing the LLM as a zero-shot classifier, using the LLM as an annotator to annotate training data, and utilizing the LLM for data augmentation. We opted for GPT3.5 and GPT4 as the LLM in the experiments due to their widespread popularity and ready accessibility within the research community.

For the supervised NLP models, we divided the data using stratified 80-20 random splits to ensure that the class distributions of the training and test set remain the same. The training set was used for training and optimization of models, and the test set was used for evaluating model performances. The same data splits were used for developing and evaluating the LLM-based approaches. The evaluation metrics were the precision, recall, and *F*_1_ score over the positive class on the test set, where *F*_1_ score was chosen as the primary metric for comparison because it ensures that neither precision (positive predictive value) nor recall (sensitivity) is optimized at the expense of the other. To reduce the risk of overfitting caused by using a single train-test split, 5-fold cross-validation was applied to evaluate the model performance. Specifically, the dataset was randomly divided into 5 equal parts. For each classification model, the model was trained and tested 5 times during cross-validation, each time using a different fold for testing and the remaining 4-folds for training. The mean and standard deviation of the evaluation metrics over the 5 test sets were reported.

#### Support vector machine

SVMs are often used when dealing with large feature spaces,^39^ making them a popular choice for text classification, including in this study. To represent the text notes as features, we used Term Frequency—Inverse Document Frequency (TF-IDF) for *n*-grams (sequences of *n*-words). In this case, 1-, 2-, 3-, and 4-grams were used. In the training process, grid search was employed to find the best values for 2 important hyperparameters: the kernel function (K) which could be either “linear” or “RBF,” and the C value.^2^^,^^4^^,^^6^^,^^8^^,^^10^ Furthermore, a class weighting strategy was implemented that automatically adjusts weights based on the inverse proportion of class frequencies in the input data, meaning the majority class receives a lower weight compared to the minority class during training, which can address data imbalance issues.

#### PLM-based classification

The model architecture for PLM-based classification contains a PLM encoder that converts the input text into a vector representation, as shown in Figure S1. Unlike the machine learning models that extract text-based features or generate *n*-grams, a PLM-based classification model splits a document into word pieces or tokens. Each token is then encoded into a vector, and these vectors are combined to form a vector representation of the document. The vector representation is then fed into a classification layer and an output layer with a softmax function. This produces a vector of equivalent size to the number of classes, from which the class with the highest value in its corresponding dimension is selected. In this study, we used 3 PLMs—RoBERTa, BERTweet, and SocBERT. These models have the same model architecture but were pretrained on different data sources. RoBERTa was pretrained on generic web text, BERTweet was pretrained on posts from Twitter only, and SocBERT was pretrained on posts from Twitter and Reddit. Hyperparameters for the 3 models and other relevant details are listed in Table S2.

#### Leveraging LLMs

##### LLM zero-shot classifier/annotator

In the context of using the LLM as an annotator, we asked the LLM to give the label of 1 sample, and the LLM responded in the way we requested. The study of how to ask the model to do this work is called prompt engineering. Because the model performance is highly dependent on the prompt, we used the same template for all classification tasks and only changed the task-specific keywords when running a task. Additionally, we asked the model to reply either 1 or 0 which indicated positive or negative class. Being generative models, the responses sometimes did not follow the instructions provided, and we considered those to be 0. The prompt template was as follows:

“You are a [*X*] system based on raw tweet data. The system should analyze the provided tweet and predict whether the user is [*X*] or not. Given a tweet as input, the system should output a 1 if the user is [*X*], and 0 otherwise. If a text response is generated, reanalyze the input until a 1 or 0 is generated.”

where [*X*] denotes the task-specific words. The detailed prompts for all classification tasks are in Table S3. After using the LLM as an annotator to annotate training data, we trained SVM and RoBERTa on the training data with the model-annotated labels. We evaluated the test data with the human-annotated labels. When utilizing the LLM as a zero-shot classification model, we leveraged the predictions generated in the experiment where the LLM served as an annotator.

##### Using LLMs for data augmentation

**Figure 2.**
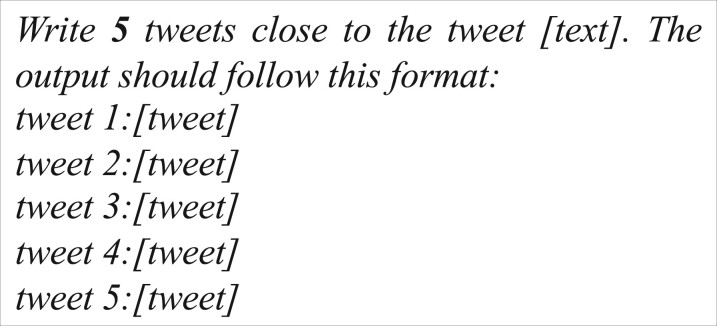
The prompt for data augmentation where [*text*] was the placeholder for the original post, and [*tweet*] was the placeholder for the post generated by the LLM.

In the realm of machine learning, it is a well-established fact that increasing the volume of data can significantly boost a model’s performance. To harness this principle, we employed an LLM to augment our training data for classification tasks. Our approach involved instructing the LLM to generate posts closely resembling each entry in our original training set. The specified prompt was structured as Figure 2. Subsequently, we assigned identical labels to the generated posts, creating a new training set that encompassed both the original posts and their LLM-generated counterparts. This expansion resulted in a training set 5 times larger than its initial size. Table S4 showcases 1 original post and its corresponding 5 generated posts. We trained the supervised NLP models on this artificially augmented dataset. Subsequent evaluation was conducted on the test data, annotated by human experts. To investigate the impact of data size on model performance, we conducted a comparative analysis of training data comprising 1, 2, 3, 4, and 5 generated posts for each original post. We conducted the experiment multiple times using all possible combinations of 1, 2, 3, and 4 posts out of the 5 posts, averaging the results to enhance performance stability. Additionally, we explored the efficacy of data augmentation by varying the percentage of augmented training data (20%, 40%, 60% 80%, and 100%) and the percentage of LLM-generated posts in the training data. This inquiry also sought to ascertain whether leveraging LLM for data augmentation could alleviate the manual effort required for data annotation.

We employed GPT3.5 and GPT4 in conducting data augmentation experiments for self-report depression and self-report COPD tasks. The rationale behind selecting these tasks was rooted in their relatively small data sizes. The cost associated with API usage for data augmentation surpassed that of classification, prompting our decision.

## Results

### Classification results

**Figure 3.**
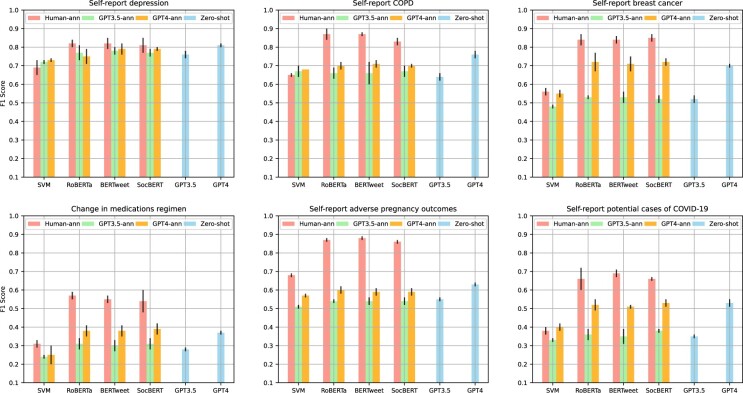
The classification results of employing human-annotated data as training data (Human-ann), utilizing LLM-annotated data as training data (GPT3.5-ann and GPT4-ann), and utilizing the LLM as a zero-shot classifier (GPT3.5 zero-shot and GPT4 zero-shot). The detailed precision, recall, and *F*_1_ scores are listed in Table S4.

Figure 3 displays the classification outcomes obtained through 3 approaches: employing human-annotated data as training data, utilizing LLM-annotated data as training data, and utilizing the LLM as a zero-shot classifier. Across all tasks, the supervised PLM-based models, namely RoBERTa, BERTweet, and SocBERT, which were trained on data annotated by humans, demonstrated superior performance compared to their counterparts that were trained on LLM-annotated data. The averaged *F*_1_ score differences for RoBERTa, BERTweet, and SocBERT trained on human-annotated data compared to their counterparts trained on GPT3.5-annotated data were 0.24 (SD: ±0.10), 0.25 (±0.11), and 0.23 (±0.11); compared to their counterparts trained on GPT4-annotated data, the differences were 0.16 (±0.07), 0.16 (±0.08), and 0.14 (±0.08), respectively. Across all tasks, the averaged *F*_1_ score difference between the top-performing model and the GPT3.5 zero-shot classifier was 0.26 (±0.11), and for the GPT4 zero-shot classifier, it was 0.15 (±0.08). However, an intriguing contrast emerged when examining the supervised SVM models. Those trained on human-annotated data exhibited inferior performance compared to their counterparts trained on GPT3.5-annotated data and GPT4-annotated data in 2 specific tasks: self-reported depression and self-reported COPD. Furthermore, the SVM model trained on GPT4-annotated data outperformed that trained on human-annotated data in the task of self-reported potential cases of COVID-19. Looking at the LLM zero-shot classifiers, the GPT3.5 zero-shot classifier outperformed SVM for a single task (self-report depression), while the GPT4 zero-shot classifier outperformed SVM in 5 out of 6 tasks (except for self-report COPD). In contrast, the PLM-based models trained on human-annotated data consistently outperformed the LLM zero-shot classifiers, while the GPT4 zero-shot classifier achieved higher *recall* than the models trained on human-annotated data in 5 out of 6 tasks (except for self-report adverse pregnancy outcomes). We also observed the differences between the 2 LLMs. Across all tasks, the GPT4 zero-shot classifier consistently delivered a higher *F*_1_ score compared to the GPT3.5 zero-shot classifier. Additionally, the supervised NLP models trained on GPT4 annotated data exhibited superior performance when compared to their counterparts trained on GPT3.5 annotated data across most tasks. These findings suggest that predictions generated by GPT4 tend to be more accurate than those from GPT3.5, which aligned with expectations given that GPT4 can be considered a more advanced version of GPT3.5.

### Data augmentation results

**Figure 4.**
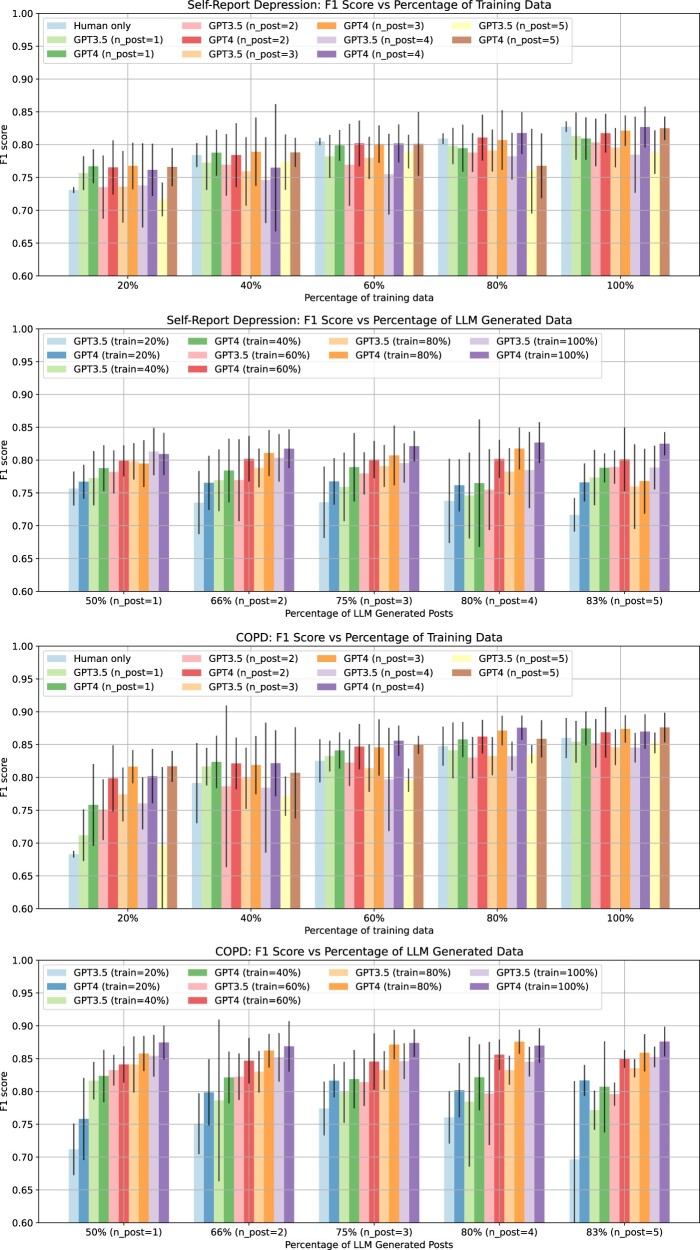
The *F*_1_ scores for the RoBERTa models trained on GPT3.5-augmented data, GPT4-augmented data, and the human-annotated data alone for self-report depression and self-report COPD, respectively.

We opted for RoBERTa as the supervised NLP model for examining the efficacy of data augmentation. This choice was motivated by the superior performance of PLM-based models over SVM. Moreover, among the 3 PLMs, RoBERTa has been subjected to more extensive research.^40–42^  Figure 4 visually represents the outcomes of RoBERTa models trained on GPT3.5 augmented data, GPT4 augmented data, and the human-annotated data for self-report depression and self-report COPD classification, respectively. Broadly, the classification performance trend across both tasks remained consistent. In comparison to models trained solely on human-annotated data, those trained on GPT4 augmented data demonstrated superior or comparable performance, especially with lower percentage of training data. However, models trained on GPT3.5-augmented data exhibited worse results when using more than 20% of training data. The optimal performance, particularly with GPT4-augmented data, was achieved when utilizing 100% of the training data for both tasks. For self-report of depression, the optimal percentage of LLM-generated posts in the training data was 83% (*n*_post = 5), and for self-report COPD, it was 80% (*n*_post = 4). For both GPT4 and GPT3.5, the models trained on varying percentages of LLM-generated posts generally exhibited wider error bars compared to those trained solely on human-annotated posts. However, this difference in error bar width appears to decrease as the percentage of training data increases, with models trained on 100% of the data showing more consistent performance across both human-annotated and LLM-generated scenarios.

## Discussion

We conducted a comprehensive comparison of classification performances by employing human-annotated data, LLM-annotated data as training data, and utilizing the LLM as a zero-shot classifier. The outcomes revealed that using LLM-annotated data only for training supervised classification models was ineffective. However, employing the LLM as a zero-shot classifier exhibited the potential to outperform traditional SVM models and achieved a higher recall than the advanced transformer-based model RoBERTa. This suggests that the LLM zero-shot classifier may in some cases serve as a valuable preprocessing module to exclude false negatives before leveraging supervised classification models. This can be particularly useful for extremely imbalanced datasets. In the context of the self-report depression classification task, we observed a marginal performance gap between the GPT4 zero-shot classifier and the PLM-based models trained on human-annotated data. This implies the promising prospect of using the LLM zero-shot classifiers to reduce the human effort required for data annotation.

In our zero-shot classification experiments, we observed that the LLMs’ outputs exhibited high sensitivity to prompt formulation. Two key issues emerged: First, the LLMs occasionally produced inconsistent responses to identical prompts when functioning as classifiers. Second, despite explicit instructions to output “1” for positive cases and “0” otherwise, the models sometimes generated responses outside this binary format. To address these challenges, we iteratively refined the prompt through minor edits and assessed its stability. Our methodology involved testing each new prompt variant on 100 randomly sampled cases from the training set, repeating this process 3 times. We ceased prompt refinement upon achieving consistent results across all 3 iterations. Our findings indicated that including the directive “If a text response is generated, reanalyze the input until a 1 or 0 is produced” significantly improved the LLMs’ adherence to the desired output format.

In the data augmentation experiments, the prompt was not fine-tuned, as it was observed that LLMs could generate data conforming to the required output format using a simple prompt without difficulty. This outcome aligns with the expectation that LLMs, given their pretraining in text generation, might exhibit superior performance in generative tasks compared to classification tasks. Our results indicated that utilizing GPT3.5 for data augmentation could potentially harm model performance. We observed that posts generated by GPT3.5 were more likely to deviate from the original content compared to those generated by GPT4. For instance, given a self-report depression post stating “Or I have depression, probably both,” GPT3.5 might generate a response like “It’s okay to not be okay sometimes. Taking the first step to seek help is brave.” While supportive, this generated content significantly differs from the original post’s tone and intent. In contrast, GPT4-generated posts consistently remained on-topic and more closely aligned with the original content. This underscores the importance of the LLM’s capability to generate high-quality posts for effective data augmentation. In contrast, data augmentation with GPT4 demonstrated improved model performances, showcasing the potential of LLMs in reducing the need for extensive training data. Additionally, while we observed that increasing the number of LLM-generated posts generally improved model performance, we did not extend our experiments beyond 5 posts. The results show that models trained on LLM-generated posts exhibit wider error bars, suggesting more variable performance compared to those trained on human-annotated data. However, this variability tends to decrease with larger training data percentages. Further research could explore whether using an even larger number of LLM-generated posts might lead to more stable or improved performance, particularly for scenarios with limited human-annotated training data. Furthermore, we found that the optimal number of augmented data and the ideal ratio of human-annotated data and LLM augmented data can be task-specific, emphasizing that increased augmented data may not lead to enhanced model performance. It remains an underexplored area and requires future investigation. We recommend researchers explore this task-specificity through grid searches to identify the most appropriate number of augmented data when applying data augmentation to a specific task. Overall, this study suggests that the best performances for text classification may be obtained by performing some manual annotations followed by data augmentation via LLMs. Note also that the LLMs we used were neither fine-tuned for social media data nor medical texts. Customizing LLMs before data augmentation may further improve performance.

### Limitations

Limitations of our study include the limited exploration of optimal prompts for enhancing LLM-based model performance, as our primary focus was on devising effective strategies for LLM integration into text classification. Future research should delve deeper into prompt engineering to ascertain its potential impact. Moreover, due to the prohibitive costs associated with utilizing proprietary models such as GPT3.5 and GPT4, we restricted our benchmarking and data augmentation experiments to a modest number of classification tasks. Consequently, our findings may not fully encapsulate the diverse range of potential applications. However, with the proliferation of open-source LLMs, future studies can extend our experiments across a wider array of datasets at a lower cost, thereby bolstering the generalizability of our conclusions.

## Conclusion

In this study, we undertook a comprehensive examination of classification performances, utilizing human-annotated data, employing the LLM as a zero-shot classifier, using the LLM as an annotator to annotate training data, and utilizing the LLM for data augmentation. Our experiments demonstrate that combining data augmentation using LLMs (GPT4) with human-annotated data to train lightweight supervised classification models achieves superior results compared to training with human-annotated data alone. Additionally, this approach outperforms zero-shot learning using GPT4 and GPT3.5. By leveraging this strategy, we can harness the power of LLMs to develop smaller, more effective domain-specific NLP models. The results indicate that using LLM-annotated data without human-annotated data for training lightweight supervised classification models is an ineffective strategy. However, LLM, as a zero-shot classifier, shows promise in excluding false negatives and potentially reducing the human effort required for data annotation. Future investigations are imperative to explore optimal training data sizes and the optimal amounts of augmented data.

## Supplementary Material

ocae210_Supplementary_Data

## Data Availability

The data underlying this article will be shared on reasonable request to the corresponding authors.
