# Supplementary material for: Evaluating large language models for health-related text classification tasks with public social media data
Source: J Am Med Inform Assoc. 2024 Aug 9;31(10):2181–9. doi: 10.1093/jamia/ocae210 (PMC11413434; doi:10.1093/jamia/ocae210)
Supplement: ocae210_Supplementary_Data [file ocae210_supplementary_data.docx]

# Supplementary Material

Figure S1: The classification model architecture of PLM-based models: A text sequence is split into a list of tokens with two special tokens, *[CLS]* and *[SEP]*, denoting the beginning and the end of the sequence, respectively. All tokens are then encoded into vector representations, known as token embeddings. After pooling, a document embedding $e_{d}$is generated and fed into a linear layer followed by a softmax activation. The output is a vector where the size corresponds to the number of classes.

Table S1: The sample positive and negative tweets for each classification tasks.

| Task | Example |
| --- | --- |
| Self-report depression | Positive:   1. I think as a result of mental strain and depression, I have lost a lot of memory. I cannot tell very normal events of my own life which happened over a span of last 2 years. It's all jumbled up and messy. And most of it seems like super imposed into a single day. 2. It being nice out makes me feel I have another life about me. Seasonal depression really hits me hard. 3. Up until <date> and the past 3 years have completely horrible but I survived it and my life is heading into a wonderful new direction with my partner and our beautiful cats. I genuinely have loved the past few months more than I have in a long time. Depression cloud is lifting 4. Just saw my Snapchat recap of the year and realised what a roller coaster this year has been. Overcoming anxiety and depression , from being fired to running an agency, from being alone to having a beautiful relationship. The only regret i have is not taking more pictures 5. The holiday season can be particularly difficult for those who struggle with depression. I couldn’t have reached the other side without the compassion of others. Reach out if you think someone needs a hand. If you are having a hard time don’t keep it to yourself. You’re not alone   Negative:   1. I'm sorry to not have posted any BJ content recently. I wanted to make christmas content, but my fiance is dealing with depression and suicidal thoughts so yea.. With his neverending work he doesn't have the time nor energy to give me a hand for the suit on a day off work. 2. Most drugs used for schizophrenia/bipolar disorders/depression are safe and do not have addictive potential I.e. there is NO RISK of developing an addiction. These drugs improve mood/psychotic symptoms. There is NO CRAVING/LOSS OF CONTROL. The outcome is usually positive. 5/n 3. how to cope with idol grad depression: watch a whole series over two days and become extremely invested in the happiness of the characters. I recommend it. now I have depression with a new side called AAAAAAAAAA LOOK AT THEY!!!! 4. it was really fun to be public about fighting fibromyalgia & depression and having a hysterectomy and have several people praise me for creating a safer work environment while knowing I was seen as a liability! 5. If you have depression or an anxiety disorder. My dms are open if you need to talk. No judgment. I'll respond as soon as i see it. |
| Self-report COPD | Positive:   \| 1. Dr. <name> is a fucking moron. He's been trying to treat my COPD. Says I don't have a lung or heart problem (made me spend all of Monday in the hospital for testing). Saying I should go back to work. Fucking idiot. I. Cannot. Fucking. Breathe. \| \| --- \| \| 1. Ok. I am going to see any many people like I say. I am not going to get the vaccine. That is my choice. I have heart disease and COPD. My doctors has not said to get it or do they encourage it. I dont know how this vaccine will work on me. \| \| 1. #CNNTownHall I am a formeformer smoker. I have COPD and chronic bronchitis. Am I at higher risk of getting Covid 19? \| \| 1. I have COPD and my right side up under my rib hurts when I breath and I cant lay on my right side at all. My chest feels tight, Ive lost my voice and starting to cough..thinking I need X-rays..what do you think? Pneumonia? \| \| 1. I am on social security and I have COPD hard to breath and my cell phone went out on me and I need help to get a cell phone is there any body out there that will help me out I have no way to call my breathing meds in and I need help to get a cell phone I am a 58 year old man \|   Negative:   \| 1. I'm fed up with able bodied people who don't know what it's like to live in care. They don't know about people who have epilepsy, autism, COPD, downs syndrome and Dementia. I'm fed up with politicians, journalists and radio presenters either slating those in care. \| \| --- \| \| 1. No stream today. My mom got her 2nd covid shot and I need to keep a close eye on her since she have COPD and other health issues. Depending on how she does next couple days I may try for a Tuesday evening stream. \| \| 1. Sooner I get an appointment for spirometry the better so I know whether I have asthma or COPD \| \| 1. Pt: I've been on warfarin for awhile. Me: Oh? What do you take it for? Pt: I have COPD. Me: Ok, but why do you take warfarin? Pt: Because of the COPD. Me: I'm not sure I'm following Pt: I'm on two of Warafin all the time. Me.....you mean oxygen? \| \| 1. I sound like I have COPD, and as a non-smoker I will be PISSED to get!! I have resigned myself to most likely getting diabetes due to obesityI deserve thatbut not a lung disease! Damn you Covid!! 5 months of this now!! \| |
| Self-report breast cancer | Positive:   \| 1. i never tried vanilla coke. i've been drinking less cola overall. i probably should cut back on coffee because of acid reflux, but i love it. i've been drinking more tea in the past few years. it's good for breast cancer survivors. \| \| --- \| \| 1. i would love to see her pull this off with my boobs lol i have so many scars from the breast cancer treatments that would be a scary sight. \| \| 1. i would be on anti-anxiety meds if i hadn’t discovered mediation after completing my breast cancer treatment \| \| 1. i have a new page called breast cancer stomp on it has a picture of me and my sister in the profile picture 2. people with breast cancer and rare diseases like mine will not be able to access the treatments. it is imperative for us to remain or gave a deal allowing us access to euratom. it’s a great worry for us. \|   Negative:   \| 1. can’t wait! tomorrow i’m selling a ton of games to get overwatch and the breast cancer mercy skin on my new pc! \| \| --- \| \| 1. i'm in a america with no patriotic outfit and my mom has breast cancer yet i have no pink clothes. but material doesn't define anything. \| \| 1. so under a certain age, no need for annual mammograms for sures. over a certain age, probably we should have annual mammograms as the risk of breast cancer increases as we age. \| \| 1. nearly fell apart at work today, and one of my older coworkers (a breast cancer survivor) helped bring me back to solid ground re: my mom. \| \| 1. i'm reading all this stuff cuz it caught my brain and i'm in bed with pneumonia and nothing else to do. she had a breast cancer scare, surgery to remove the cyst, non-malignant, then two positive cervical biopsies for malignant cells followed by leep procedures. \| |
| Change in medications regimen | Positive:   \| 1. i just can't handle another interrogation. i saw covering dr. got the same question why are you here. i have to make an appointment with regular dr for plavix and atenelol. odd, platelets down without it, but bleed easily. stopped crestor too, but numbers are high, but better. \| \| --- \| \| 1. thank you cortisone shots for bruising the hell out of my foot. \| \| 1. at the dentist yet again. thank goodness for novicane! \| \| 1. accidentally took ibuprofen thinking it was my allergies pills, keep in mind i'm also allergic to ibuprofen \| \| 1. brad took the last of my tums. so now i have heartburn and nothing to get rid of it. \|   Negative:   \| 1. after labor i thought the pain would be gone, but its so much worse after labor ­popping the percs they prescribed me like no tomorrow \| \| --- \| \| 1. my doctor prescribed me oxy. it's going to have me high as shit. \| \| 1. man, like, i started taking robitussin & benadryl last night and it has worked a miracle in my life. \| \| 1. apple juice and grape juice are basically laxatives lol but seriously \| \| 1. about to buy some tums already \| |
| Self-report adverse pregnancy outcomes | Positive:   \| 1. people say that babies are ugly when they just come out the vagina but my lil girl was seriously the cutest n so perfect n she was premature \| \| --- \| \| 1. because we didn't know the reasons for my miscarriages, i always assumed it was something i did. something i could of done differently. \| \| 1. i'm truly blessed with this pregnancy and my healthy baby boy. actually i'm beyond blessed! my rainbow baby! \| \| 1. my baby is in the nicu and i hate it \| \| 1. i wish it was easier to talk about miscarriages. it's so taboo. people whisper about it, but no one really talks about it. \|   Negative:   \| 1. howling my eyes out at corrie. having a stillborn must be the worst thing in the world \| \| --- \| \| 1. and after having a year where so many women i know personally had miscarriages or stillborns, i'm hypersensitive about it. \| \| 1. one of my friends is pregnant with her rainbow baby and i'm so beyond happy for her that i can't stop crying. \| \| 1. i remember when he was just a couple weeks old he was such a tiny little baby i had to buy him premature outfits \| \| 1. when i read heart breaking stories about people miscarrying or having still born babies, i can't even imagine someone pretending to be \| |
| Self-report potential cases of COVID-19 | Positive:   \| 1. i have a sore throat, headache and stuffy nose. am i sick or do i have coronavirus \| \| --- \| \| 1. when people be coughing around me it just give me coronavirus vibes \| \| 1. life is short mang! i was just cooling on tuesday with no issue then yesterday I’m in the hospital with the flu, dehydration, a fever and my blood pressure was a lil high. all of it was just random. my brother joked that i had that coronavirus but seriously gotta take it easy. \| \| 1. so much coughing at madrid airport, I’ll be lucky to leave without ebola let alone coronavirus \| \| 1. confirmed case of coronavirus in the hospital i work in \|   Negative:   \| 1. where when how and why did this disease coronavirus came out of how did it start coronavirus is gonna get us sick and i may die from that disease i was thinking the coronavirus is coming from the corona beers \| \| --- \| \| 1. someone ought to cough in trump's face and i hope the coronavirus is listening. \| \| 1. coronavirus in mexico during spring break? someone quarantine us here \| \| 1. wow, my job is considering making us work from home until further notice due to coronavirus. I’m here for it. ima miss the homies though. \| \| 1. so coronavirus has hit my area. was only a matter of time. just a wait now to see how many people the gentleman has come into contact with already. soon be time for self quarantine in the mcleans. wonder if my boss will be so understanding when i tell him my plans \| |

Table S2: The full hyperparameter configurations of the RoBERTa classification model.

| **Hyper-parameter** | **Value** |
| --- | --- |
| Number of Layers | 12 |
| Hidden size | 768 |
| FFN inner hidden size | 3072 |
| Attention heads | 12 |
| Attention head size | 64 |
| Dropout | 0.1 |
| Attention Dropout | 0.1 |
| Weight Decay | 0.01 |
| Learning Rate Decay | Linear |
| Adam $\beta_{1}$ | 0.9 |
| Adam $\beta_{2}$ | 0.98 |
| Gradient Clipping | 0 |
| Batch Size | 32 |
| Warmup Ratio | 0 |
| Adam Epsilon | $1\times{10}^{-8}$ |

Table S3: The full prompt for each classification task.

| Task | Prompt |
| --- | --- |
| Self-report depression | You are a depression detection system based on raw tweet data. The system should analyze the provided tweet and predict whether the user is self-reporting depression or not. Given a tweet as input, the system should output a 1 if the user is self-reporting depression, and 0 otherwise. If a text response is generated, reanalyze the input until a 1 or 0 is generated. |
| Self-report COPD | You are a Chronic Obstructive Pulmonary Disease (COPD) detection system based on raw tweet data. The system should analyze the provided tweet and predict whether the user is self-reporting COPD or not. Given a tweet as input, the system should output a 1 if the user is self-reporting COPD, and 0 otherwise. If a text response is generated, reanalyze the input until a 1 or 0 is generated. |
| Self-report breast cancer | You are a breast cancer detection system based on raw tweet data. The system should analyze the provided tweet and predict whether the user is self-reporting breast cancer or not. Given a tweet as input, the system should output a 1 if the user is self-reporting breast cancer, and 0 otherwise. If a text response is generated, reanalyze the input until a 1 or 0 is generated. |
| Change in medications regimen | You are a change in medication regimen detection system based on social media posts. The system should analyze the provided post and predict whether the user is describing a change in medication regimen or not. Given a post as input, the system should output a 1 if the user is describing a change in medication regimen, and 0 otherwise. If a text response is generated, reanalyze the input until a 1 or 0 is generated. |
| Self-report adverse pregnancy outcomes | You are an adverse pregnancy outcome detection system based on social media posts. The system should analyze the provided post and predict whether the user is self-reporting an adverse pregnancy outcome or not. Given a post as input, the system should output a 1 if the user is self-reporting an adverse pregnancy outcome, and 0 otherwise. If a text response is generated, reanalyze the input until a 1 or 0 is generated. |
| Self-report potential cases of COVID-19 | You are an COVID-19 detection system based on social media posts. The system should analyze the provided post and predict whether the user is self-reporting a potential case of COVID-19 or not. Given a post as input, the system should output a 1 if the user is self-reporting a potential case of COVID-19, and 0 otherwise. If a text response is generated, reanalyze the input until a 1 or 0 is generated. |

Table S4: The showcase of data augmentation with GPT4 for self-report depression.

| Source | Post |
| --- | --- |
| Original | SSRIs have never done much for me’ I've been medication-free for 3 years. I cope well at times, am dysfunctional other times. I hope to be more consistently regulated. I also hope to have fewer intrusive thoughts/memories, and reduce anxiety and depression symptoms. |
| Generated by GPT4 | 3 years off SSRIs and still counting. My journey has highs and lows but I'm striving for balance. Here's to fewer anxiety episodes and quieter minds. #MentalHealthJourney |
|  | Sometimes I handle life well, other times not so much. Been off meds for 3 years. Looking forward to a future where my mind is calmer, less anxious, and less haunted by the past. #HealingJourney |
|  | 3 years of being med-free has its share of triumphs and struggles. I yearn for steadier emotions, less anxiety and depression, and a mind free of unwanted thoughts. #MentalHealthAwareness |
|  | Three years off meds and still fighting. Some days are easier than others. Here's to hoping for a future with less anxiety, fewer intrusive thoughts, and more consistency. #MentalWellness |
|  | Been off SSRIs for 3 years now. The journey is bumpy but I'm hopeful for more regulated emotions, less anxiety, and a mind free from recurring distressing memories. #MentalHealth |

Table S5: The precision, recall, and F_1_ scores of employing human-annotated data as training data (Human-ann), utilizing LLM-annotated data as training data (GPT3.5-ann and GPT4-ann), and utilizing the LLM as a zero-shot classifier (GPT3.5 zero-shot and GPT4 zero-shot).

| **Model** | **Label source** | **Precision** | **Recall** | **F1** |
| --- | --- | --- | --- | --- |
| Self-report depression | | | | |
| SVM | Human-ann | 0.68 (±0.01) | 0.70 (±0.07) | 0.69 (±0.04) |
| SVM | GPT3.5-ann | 0.58 (±0.02) | 0.97 (±0.08) | 0.72 (±0.01) |
| SVM | GPT4-ann | 0.58 (±0.02) | 0.99 (±0.02) | 0.73 (±0.01) |
| RoBERTa | Human-ann | 0.76 (±0.05) | 0.88 (±0.07) | **0.82 (±0.02)** |
| RoBERTa | GPT3.5-ann | 0.66 (±0.07) | 0.94 (±0.08) | 0.77 (±0.04) |
| RoBERTa | GPT4-ann | 0.61 (±0.05) | **0.99 (±0.01)** | 0.75 (±0.04) |
| BERTweet | Human-ann | **0.80 (±0.06)** | 0.85 (±0.08) | **0.82 (±0.03)** |
| BERTweet | GPT3.5-ann | 0.70 (±0.06) | 0.88 (±0.10) | 0.78 (±0.02) |
| BERTweet | GPT4-ann | 0.66 (±0.05) | 0.98 (±0.01) | 0.79 (±0.03) |
| SocBERT | Human-ann | 0.78 (±0.03) | 0.85 (±0.07) | 0.81 (±0.04) |
| SocBERT | GPT3.5-ann | 0.68 (±0.05) | 0.88 (±0.05) | 0.77 (±0.02) |
| SocBERT | GPT4-ann | 0.66 (±0.03) | 0.97 (±0.02) | 0.79 (±0.01) |
| GPT3.5 zero-shot | | 0.70 (±0.01) | 0.84 (±0.05) | 0.76 (±0.02) |
| GPT4 zero-shot | | 0.69 (±0.01) | 0.98 (±0.01) | 0.81 (±0.01) |
| Self-report COPD | | | | |
| SVM | Human-ann | 0.65 (±0.02) | 0.65 (±0.03) | 0.65 (±0.01) |
| SVM | GPT3.5-ann | 0.52 (±0.00) | 0.95 (±0.12) | 0.67 (±0.03) |
| SVM | GPT4-ann | 0.52 (±0.00) | 1.00 (±0.00) | 0.68 (±0.00) |
| RoBERTa | Human-ann | 0.84 (±0.04) | 0.91 (±0.05) | **0.87 (±0.03)** |
| RoBERTa | GPT3.5-ann | 0.53 (±0.02) | 0.90 (±0.15) | 0.66 (±0.03) |
| RoBERTa | GPT4-ann | 0.54 (±0.03) | 0.99 (±0.01) | 0.70 (±0.02) |
| BERTweet | Human-ann | **0.87 (±0.05)** | 0.87 (±0.04) | **0.87 (±0.01)** |
| BERTweet | GPT3.5-ann | 0.53 (±0.02) | 0.90 (±0.20) | 0.66 (±0.06) |
| BERTweet | GPT4-ann | 0.56 (±0.03) | 0.97 (±0.03) | 0.71 (±0.02) |
| SocBERT | Human-ann | 0.80 (±0.03) | 0.87 (±0.07) | 0.83 (±0.02) |
| SocBERT | GPT3.5-ann | 0.54 (±0.02) | 0.90 (±0.10) | 0.67 (±0.03) |
| SocBERT | GPT4-ann | 0.54 (±0.01) | **1.00 (±0.01)** | 0.70 (±0.01) |
| GPT3.5 zero-shot | | 0.56 (±0.02) | 0.74 (±0.03) | 0.64 (±0.02) |
| GPT4 zero-shot | | 0.61 (±0.03) | 0.99 (±0.01) | 0.76 (±0.02) |
| Self-report breast cancer | | | | |
| SVM | Human-ann | 0.53 (±0.02) | 0.70 (±0.02) | 0.60 (±0.01) |
| SVM | GPT3.5-ann | 0.36 (±0.01) | 0.74 (±0.03) | 0.48 (±0.02) |
| SVM | GPT4-ann | 0.42 (±0.01) | 0.76 (±0.04) | 0.54 (±0.02) |
| RoBERTa | Human-ann | 0.80 (±0.04) | 0.88 (±0.02) | 0.84 (±0.03) |
| RoBERTa | GPT3.5-ann | 0.38 (±0.01) | 0.88 (±0.06) | 0.53 (±0.01) |
| RoBERTa | GPT4-ann | 0.59 (±0.07) | 0.91 (±0.04) | 0.72 (±0.05) |
| BERTweet | Human-ann | 0.81 (±0.04) | 0.87 (±0.03) | 0.84 (±0.02) |
| BERTweet | GPT3.5-ann | 0.38 (±0.01) | 0.87 (±0.08) | 0.53 (±0.03) |
| BERTweet | GPT4-ann | 0.59 (±0.08) | 0.89 (±0.06) | 0.71 (±0.04) |
| SocBERT | Human-ann | **0.84 (±0.03)** | 0.85 (±0.01) | **0.85 (±0.02)** |
| SocBERT | GPT3.5-ann | 0.38 (±0.03) | 0.85 (±0.06) | 0.52 (±0.02) |
| SocBERT | GPT4-ann | 0.59 (±0.03) | 0.90 (±0.04) | 0.72 (±0.02) |
| GPT3.5 zero-shot | | 0.37 (±0.02) | 0.86 (±0.04) | 0.52 (±0.02) |
| GPT4 zero-shot | | 0.56 (±0.01) | **0.95 (±0.01)** | 0.70 (±0.01) |
| Change in medications regimen | | | | |
| SVM | Human-ann | 0.23 (±0.02) | 0.49 (±0.03) | 0.31 (±0.02) |
| SVM | GPT3.5-ann | 0.14 (±0.00) | 0.74 (±0.03) | 0.24 (±0.01) |
| SVM | GPT4-ann | 0.15 (±0.03) | 0.80 (±0.11) | 0.25 (±0.05) |
| RoBERTa | Human-ann | 0.54 (±0.12) | 0.63 (±0.10) | **0.57 (±0.02)** |
| RoBERTa | GPT3.5-ann | 0.19 (±0.02) | 0.79 (±0.11) | 0.31 (±0.03) |
| RoBERTa | GPT4-ann | 0.26 (±0.03) | 0.75 (±0.06) | 0.38 (±0.03) |
| BERTweet | Human-ann | **0.67 (±0.11)** | 0.48 (±0.08) | 0.55 (±0.02) |
| BERTweet | GPT3.5-ann | 0.18 (±0.02) | 0.83 (±0.09) | 0.30 (±0.03) |
| BERTweet | GPT4-ann | 0.26 (±0.03) | 0.73 (±0.10) | 0.38 (±0.03) |
| SocBERT | Human-ann | 0.58 (±0.08) | 0.52 (±0.08) | 0.54 (±0.06) |
| SocBERT | GPT3.5-ann | 0.19 (±0.03) | **0.85 (±0.07)** | 0.31 (±0.03) |
| SocBERT | GPT4-ann | 0.28 (±0.03) | 0.68 (±0.09) | 0.39 (±0.03) |
| GPT3.5 zero-shot | | 0.17 (±0.00) | 0.75 (±0.02) | 0.28 (±0.01) |
| GPT4 zero-shot | | 0.24 (±0.01) | 0.77 (±0.05) | 0.37 (±0.01) |
| Self-report adverse pregnancy outcomes | | | | |
| SVM | Human-ann | 0.70 (±0.01) | 0.67 (±0.01) | 0.68 (±0.01) |
| SVM | GPT3.5-ann | 0.44 (±0.01) | 0.62 (±0.02) | 0.51 (±0.01) |
| SVM | GPT4-ann | 0.45 (±0.01) | 0.78 (±0.02) | 0.57 (±0.01) |
| RoBERTa | Human-ann | 0.82 (±0.04) | **0.92 (±0.03)** | 0.87 (±0.01) |
| RoBERTa | GPT3.5-ann | 0.46 (±0.02) | 0.65 (±0.05) | 0.54 (±0.01) |
| RoBERTa | GPT4-ann | 0.49 (±0.02) | 0.77 (±0.06) | 0.60 (±0.02) |
| BERTweet | Human-ann | **0.88 (±0.03)** | 0.89 (±0.03) | **0.88 (±0.01)** |
| BERTweet | GPT3.5-ann | 0.46 (±0.03) | 0.65 (±0.04) | 0.54 (±0.02) |
| BERTweet | GPT4-ann | 0.49 (±0.01) | 0.75 (±0.04) | 0.59 (±0.02) |
| SocBERT | Human-ann | 0.85 (±0.03) | 0.87 (±0.03) | 0.86 (±0.01) |
| SocBERT | GPT3.5-ann | 0.46 (±0.01) | 0.65 (±0.05) | 0.54 (±0.02) |
| SocBERT | GPT4-ann | 0.48 (±0.02) | 0.77 (±0.02) | 0.59 (±0.02) |
| GPT3.5 zero-shot | | 0.49 (±0.01) | 0.62 (±0.02) | 0.55 (±0.01) |
| GPT4 zero-shot | | 0.52 (±0.01) | 0.81 (±0.01) | 0.63 (±0.01) |
| Self-report potential cases of COVID-19 | | | | |
| SVM | Human-ann | 0.59 (±0.05) | 0.28 (±0.03) | 0.38 (±0.02) |
| SVM | GPT3.5-ann | 0.21 (±0.01) | 0.71 (±0.03) | 0.33 (±0.01) |
| SVM | GPT4-ann | 0.43 (±0.02) | 0.38 (±0.03) | 0.40 (±0.02) |
| RoBERTa | Human-ann | **0.68 (±0.07)** | 0.64 (±0.11) | 0.66 (±0.06) |
| RoBERTa | GPT3.5-ann | 0.23 (±0.03) | **0.86 (±0.07)** | 0.36 (±0.03) |
| RoBERTa | GPT4-ann | 0.48 (±0.06) | 0.60 (±0.09) | 0.52 (±0.03) |
| BERTweet | Human-ann | **0.68 (±0.06)** | 0.71 (±0.07) | **0.69 (±0.02)** |
| BERTweet | GPT3.5-ann | 0.23 (±0.03) | 0.79 (±0.13) | 0.35 (±0.04) |
| BERTweet | GPT4-ann | 0.43 (±0.03) | 0.61 (±0.02) | 0.51 (±0.01) |
| SocBERT | Human-ann | 0.66 (±0.06) | 0.67 (±0.06) | 0.66 (±0.01) |
| SocBERT | GPT3.5-ann | 0.24 (±0.01) | 0.83 (±0.05) | 0.38 (±0.01) |
| SocBERT | GPT4-ann | 0.49 (±0.02) | 0.57 (±0.04) | 0.53 (±0.02) |
| GPT3.5 zero-shot | | 0.23 (±0.01) | 0.74 (±0.03) | 0.35 (±0.01) |
| GPT4 zero-shot | | 0.45 (±0.02) | 0.65 (±0.03) | 0.53 (±0.02) |

Table S6: The F_1_ scores of RoBERTa trained on human annotated posts, GPT3.5 annotated posts, and GPT4 annotated posts with various percentage of training data and various percentage of LLM generated posts.

| n_post | Percent of training data | F_1_ score | Standard deviation |
| --- | --- | --- | --- |
| Self-report depression (human annotated posts) | | | |
| 0 | 20 | 0.731 | 0.004 |
| 0 | 40 | 0.784 | 0.018 |
| 0 | 60 | 0.805 | 0.005 |
| 0 | 80 | 0.809 | 0.008 |
| 0 | 100 | 0.827 | 0.008 |
| Self-report depression (GPT3.5 generated posts) | | | |
| 1 | 20 | 0.757 | 0.026 |
| 2 | 20 | 0.735 | 0.048 |
| 3 | 20 | 0.736 | 0.054 |
| 4 | 20 | 0.738 | 0.064 |
| 5 | 20 | 0.717 | 0.026 |
| 1 | 40 | 0.772 | 0.041 |
| 2 | 40 | 0.769 | 0.047 |
| 3 | 40 | 0.759 | 0.052 |
| 4 | 40 | 0.746 | 0.065 |
| 5 | 40 | 0.773 | 0.042 |
| 1 | 60 | 0.782 | 0.033 |
| 2 | 60 | 0.769 | 0.062 |
| 3 | 60 | 0.780 | 0.032 |
| 4 | 60 | 0.755 | 0.061 |
| 5 | 60 | 0.789 | 0.026 |
| 1 | 80 | 0.798 | 0.028 |
| 2 | 80 | 0.788 | 0.029 |
| 3 | 80 | 0.791 | 0.032 |
| 4 | 80 | 0.782 | 0.036 |
| 5 | 80 | 0.760 | 0.064 |
| 1 | 100 | 0.813 | 0.036 |
| 2 | 100 | 0.803 | 0.036 |
| 3 | 100 | 0.796 | 0.030 |
| 4 | 100 | 0.785 | 0.058 |
| 5 | 100 | 0.789 | 0.033 |
| Self-report depression (GPT4 generated posts) | | | |
| 1 | 20 | 0.767 | 0.026 |
| 2 | 20 | 0.765 | 0.041 |
| 3 | 20 | 0.768 | 0.035 |
| 4 | 20 | 0.762 | 0.040 |
| 5 | 20 | 0.766 | 0.029 |
| 1 | 40 | 0.788 | 0.035 |
| 2 | 40 | 0.784 | 0.049 |
| 3 | 40 | 0.789 | 0.052 |
| 4 | 40 | 0.765 | 0.097 |
| 5 | 40 | 0.788 | 0.022 |
| 1 | 60 | 0.799 | 0.024 |
| 2 | 60 | 0.802 | 0.035 |
| 3 | 60 | 0.801 | 0.028 |
| 4 | 60 | 0.802 | 0.029 |
| 5 | 60 | 0.801 | 0.048 |
| 1 | 80 | 0.795 | 0.036 |
| 2 | 80 | 0.811 | 0.035 |
| 3 | 80 | 0.807 | 0.045 |
| 4 | 80 | 0.818 | 0.032 |
| 5 | 80 | 0.768 | 0.049 |
| 1 | 100 | 0.809 | 0.032 |
| 2 | 100 | 0.817 | 0.029 |
| 3 | 100 | 0.821 | 0.023 |
| 4 | 100 | 0.827 | 0.031 |
| 5 | 100 | 0.825 | 0.018 |
| COPD (human annotated posts) | | | |
| 0 | 20 | 0.683 | 0.005 |
| 0 | 40 | 0.791 | 0.061 |
| 0 | 60 | 0.825 | 0.033 |
| 0 | 80 | 0.848 | 0.030 |
| 0 | 100 | 0.860 | 0.031 |
| COPD (GPT3.5 generated posts) | | | |
| 1 | 20 | 0.712 | 0.039 |
| 2 | 20 | 0.751 | 0.046 |
| 3 | 20 | 0.774 | 0.041 |
| 4 | 20 | 0.760 | 0.040 |
| 5 | 20 | 0.697 | 0.119 |
| 1 | 40 | 0.816 | 0.029 |
| 2 | 40 | 0.786 | 0.123 |
| 3 | 40 | 0.799 | 0.046 |
| 4 | 40 | 0.784 | 0.099 |
| 5 | 40 | 0.771 | 0.030 |
| 1 | 60 | 0.832 | 0.023 |
| 2 | 60 | 0.823 | 0.035 |
| 3 | 60 | 0.814 | 0.036 |
| 4 | 60 | 0.797 | 0.079 |
| 5 | 60 | 0.796 | 0.018 |
| 1 | 80 | 0.841 | 0.043 |
| 2 | 80 | 0.830 | 0.032 |
| 3 | 80 | 0.832 | 0.029 |
| 4 | 80 | 0.832 | 0.022 |
| 5 | 80 | 0.835 | 0.014 |
| 1 | 100 | 0.854 | 0.032 |
| 2 | 100 | 0.852 | 0.037 |
| 3 | 100 | 0.846 | 0.027 |
| 4 | 100 | 0.845 | 0.023 |
| 5 | 100 | 0.853 | 0.016 |
| COPD (GPT4 generated posts) | | | |
| 1 | 20 | 0.758 | 0.062 |
| 2 | 20 | 0.799 | 0.051 |
| 3 | 20 | 0.816 | 0.025 |
| 4 | 20 | 0.802 | 0.041 |
| 5 | 20 | 0.817 | 0.024 |
| 1 | 40 | 0.824 | 0.040 |
| 2 | 40 | 0.821 | 0.039 |
| 3 | 40 | 0.819 | 0.044 |
| 4 | 40 | 0.822 | 0.050 |
| 5 | 40 | 0.807 | 0.069 |
| 1 | 60 | 0.841 | 0.028 |
| 2 | 60 | 0.847 | 0.035 |
| 3 | 60 | 0.846 | 0.043 |
| 4 | 60 | 0.856 | 0.023 |
| 5 | 60 | 0.850 | 0.014 |
| 1 | 80 | 0.858 | 0.027 |
| 2 | 80 | 0.862 | 0.026 |
| 3 | 80 | 0.871 | 0.022 |
| 4 | 80 | 0.876 | 0.018 |
| 5 | 80 | 0.859 | 0.028 |
| 1 | 100 | 0.875 | 0.026 |
| 2 | 100 | 0.869 | 0.038 |
| 3 | 100 | 0.874 | 0.021 |
| 4 | 100 | 0.870 | 0.026 |
| 5 | 100 | 0.876 | 0.023 |
